# Vitamin D supplementation improves anxiety but not depression symptoms in patients with vitamin D deficiency

**DOI:** 10.1002/brb3.1760

**Published:** 2020-09-18

**Authors:** Cuizhen Zhu, Yu Zhang, Ting Wang, Yezhe Lin, Jiakuai Yu, Qingrong Xia, Peng Zhu, Dao‐min Zhu

**Affiliations:** ^1^ Department of Sleep Disorders Affiliated Psychological Hospital of Anhui Medical University Hefei China; ^2^ Hefei Fourth People's Hospital Hefei China; ^3^ Anhui Mental Health Center Hefei China; ^4^ Department of Psychiatry University of Rochester Medical Center Rochester NY USA; ^5^ Department of Maternal, Child & Adolescent Health School of Public Health Anhui Medical University Hefei China

**Keywords:** anxiety, depression, vitamin D deficiency, vitamin D supplementation

## Abstract

**Objective:**

Epidemiological evidence indicated a relationship between vitamin D (VD) and depression with anxiety, but their therapeutic relationship has not been fully elucidated. This study aimed to examine whether VD supplementation would relieve symptoms in patients with depression and anxiety with low serum 25‐hydroxy VD [25(OH) D] levels.

**Method:**

Participants with low 25(OH)D levels were randomized to control or daily VD group and were followed up for 6 months. Serum concentrations of 25(OH) D were measured using commercial kits. Psychological symptoms were evaluated with the Hamilton Depression Rating Scale‐17 (HAMD‐17), Revised Social Anhedonia Scale (RSAS), Revised Physical Anhedonia scale (RPAS), and Hamilton Anxiety Rating Scale‐14 (HAMA‐14). The trial was listed in the trial registration (http://www.medresman.org.cn/uc/index.aspx; NTR number: ChiCTR2000030130).

**Results:**

In this clinical population, no significant difference in depression symptoms was detected between VD group and control group at both baseline and at the endpoint of our study. The HAMD‐17, RSAS, and RPAS scores did not change significantly between VD and control groups from baseline to endpoint (all *p* > .05). However, there was a significant difference in time effect of the total HAMA‐14 scores between the two groups (*β* [95% Cl] = −2.235 [−3.818, −0.653], *p* = .006).

**Conclusions:**

Vitamin D supplementation could improve the anxiety symptoms but not depressive symptoms in depressive patients with low VD level after the 6‐month intervention.

## Graphical abstract

Vitamin D (VD) supplementation could improve the anxiety symptoms but not depressive symptoms in depressive patients with low VD level after the 6‐month intervention.

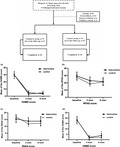

## 1 INTRODUCTION

Depression and anxiety are two of the most common debilitating illnesses among all mental disorders. Approximately 322 million people suffered from depression worldwide, leading depression to the major cause of disability over the globe (7.5% of all years lived with disability [YLDs]) (Amos et al., 2018; Li et al., 2019). As evidenced by epidemiological and clinical studies, in developed countries, 35.5%–50.3% of patients with serious mood disorders did not receive appropriate treatment and the rate rocketed to 76.3%–85.4% in less developed countries (Dean & Keshavan, 2017). Multiple hypotheses regarding possible pathophysiological mechanisms of these mood disorders included altered neurotransmission, chemical imbalance in the brain, inflammation, reduced neuroplasticity, chronic stress, and nutrient deficiency (Berridge, 2017; Maletic et al., 2007; Pertile, Cui, Hammond, & Eyles, 2018). Extensive research over the past decades has shown a critical role of malnutrition in depression and anxiety disorder (Kuygun Karci & Gul Celik, 2020; Milaneschi et al., 2019; Sanchez‐Villegas et al., 2009). Moreover, this body of research has supported a complex involvement of vitamin D (VD) deficiency in mood disorders (Ju, Lee, & Jeong, 2013); however, the relationship between the two was not fully elucidated. While some studies addressed the benefits of VD supplementation on depressive and anxiety symptoms, recent randomized trials presented mixed results (Jorde & Grimnes, 2019; Lansdowne & Provost 1998; Vieth, Kimball, Hu, & Walfish, 2004).

To understand the pathophysiology relationship between mood disorders and VD, it is necessary to explore how VD functions. The active form of VD is 1, 25‐dihydroxy vitamin D 3 [1, 25(OH) 2 D3], which is formed by a series of reactions that take place in a number of different tissues. The VD3 enters the blood and is transferred to the liver where a hydroxyl group is added to the C‐25 position by a vitamin D 25‐hydroxylase to form 25‐hydroxyvitamin D 3 [25(OH)D 3] that is the immediate precursor for active vitamin D. 25‐hydroxy vitamin D [25(OH)D] is the main circulating metabolite of vitamin D, and the serum 25(OH)D level is the most reliable marker of vitamin D status (Almeida, Hankey, Yeap, Golledge, & Flicker, 2015; Jamilian, Bagherzadeh, Nazeri, & Hassanijirdehi, 2013). Rodent experiments have demonstrated that active VD enhanced glutamate and glutamine metabolism in neurons, and therefore participated in behavioral changes and neurotransmitter‐level alteration (Harms et al., 2012; Michael et al., 2007). Several studies also indicated that VD may be relevant to the gene expression of neurotrophic factors, which consequently stimulated neurogenesis and promoted antioxidation in neurons that protected them from oxidative degenerative processes (Czaja & Montano‐Loza, 2019; Gezen‐Ak, Dursun, & Yilmazer, 2013; Jeon & Shin, 2018; Khairy & Attia, 2019). Furthermore, studies have demonstrated VD as an essential coenzyme in the synthesis of monoamines such as norepinephrine and dopamine (Chester et al., 2015; Cui, Pertile, Liu, & Eyles, 2015; Pertile, Cui, & Eyles, 2016; Pertile et al., 2018). Those pieces of evidence above indicate the role of VD as a critical neurosteroid hormone involved in numerous brain processes, including brain neuroplasticity, neuroimmunomodulation, and brain development which could have an indirect impact on mood regulation.

The relationship between depression and VD has been reported in some population‐based studies; however, some other studies revealed contradicted results (Bolland, Grey, Gamble, & Reid, 2011; de Koning et al., 2019). Especially, after regressing for confounding factors, including geography, body mass index, physical activity, and smoking, the relationship between VD and depression did not pass the significance in some studies. Notwithstanding, in cross‐sectional and randomized placebo‐controlled studies, some have shown a significant effect of VD supplementation on depressive symptoms, whereas others found no such associations (Jorde & Kubiak, 2018). In addition, depression with higher severity often tied with more anxiety symptoms, although the risk factors for the two conditions overlapped upon each other, they are still distinct entity (Shen et al., 2008). Studies exploring the association of VD levels and anxiety are still scarce and underinvestigated, as VD deficiency is potentially preventable for depression with anxiety symptoms, more studies are needed to explore the causal relationship between VD status and symptoms of depression and anxiety.

To the best of our knowledge, it is still unclear whether a decreased serum VD level is associated with anxiety independently from depression. Therefore, we performed a longitudinal study hypothesizing that the more severe depressive and anxiety symptoms would be related to lower VD serum and vice versa. In this study, we included a sample of participants with depression with anxiety; to investigate the VD supplements can effectively improve the symptoms of depression with anxiety patients.

## 2 METHODS AND MATERIALS

### 2.1 Design

The project is a clinical trial to study the effects of VD supplementation on depression with tied anxiety symptoms. This study was approved by the Medical Ethics Committee of Anhui mental health center (AMHC). All participants were provided written informed consent under the tenets of the Declaration of Helsinki.

Patients were recruited through advertisements from AMHC in China from November 2015 to March 2019. All patients were assessed by the Mini‐International Neuropsychiatric Interview (MINI) 6.0.0 (Kadri et al., 2005; Sheehan et al., 1997) to confirm the diagnosis of major depressive disorders (MDD) (Sheehan, Harnett‐Sheehan, Spann, Thompson, & Prakash, 2011). Sociodemographic data form, Hamilton Depression Rating Scale‐17 (HAMD‐17), Revised Social Anhedonia Scale (RSAS), Revised Physical Anhedonia scale (RPAS), and Hamilton Anxiety Rating Scale‐14 (HAMA‐14), was applied to all participants. In our study, HAMD‐17 was used to determine the severity of depressive symptoms. RSAS and RPAS were recommended optionally to determine the level of depression in the presence of social withdrawal and somatization symptoms.

The inclusion criteria for depressive patients were as follows: (a) diagnosis of MDD according to Diagnosis and Statistical Manual—5th edition (DSM‐V); (b) age 18–60 years; (c) Han Chinese ethnicity; and (d) serum 25(OH) D levels ≤75 nmol/L before the entry of our study. The exclusion criteria were as follows: other concurrent psychiatric disorders defined in the DSM‐V such as schizophrenia, and substance use disorders; and current severe physical conditions (e.g., neurological diseases, malignancy, cardiovascular diseases, respiratory diseases, severe trauma, state of pregnancy or breastfeeding).

**FIGURE 1:**
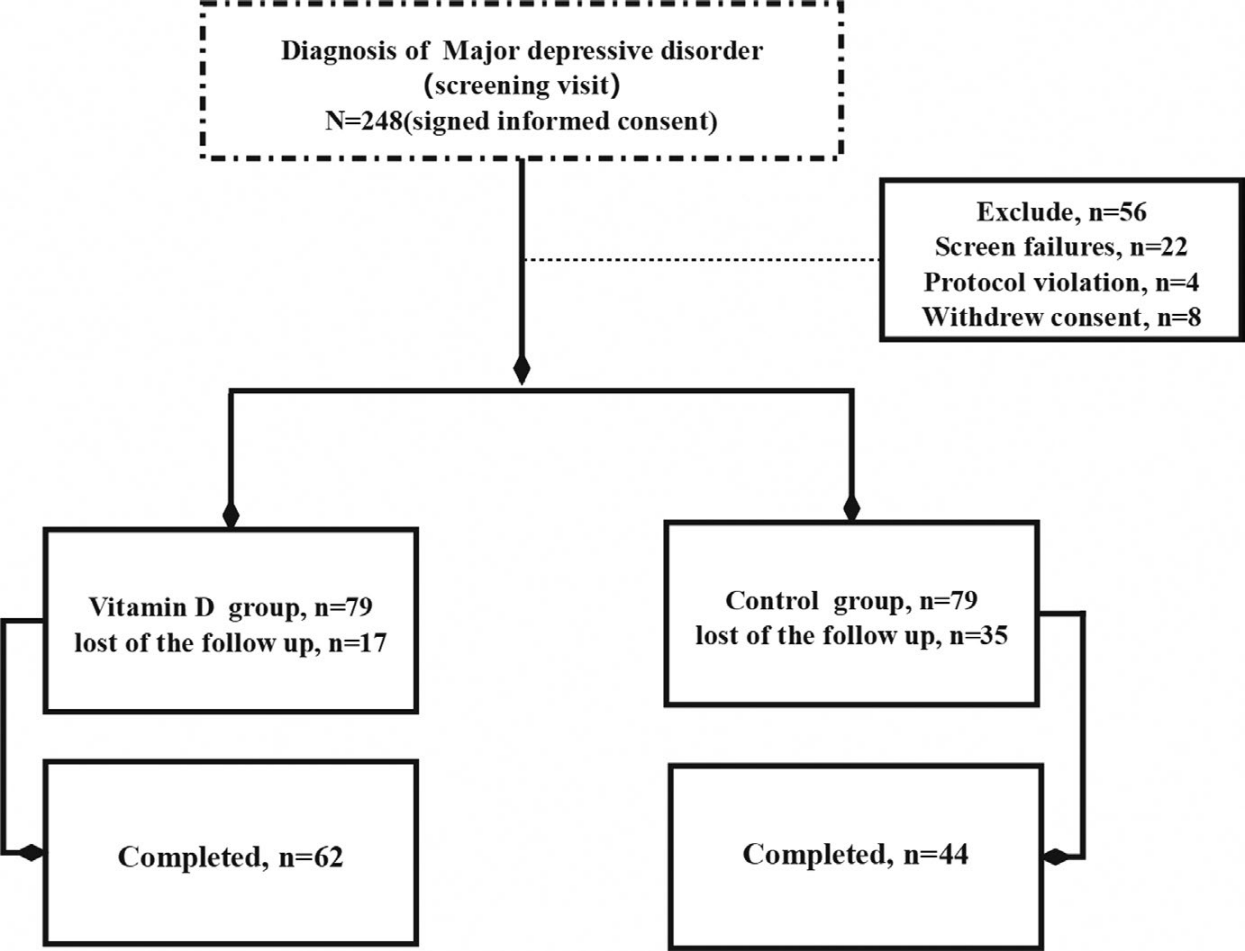
Study selection processes

After the strict clinical screening, a sample of 248 subjects entered the criteria of our study, among whom 158 people agreed to participate in this experiment with written informed consent. At the baseline of our study, subjects with serum 25(OH) D levels ≤75 nmol/L were randomly assigned to the intervention group (*n* = 79) and control group (*n* = 79). All participants were followed up in the 3rd and 6th months. 17 patients in the intervention group and 35 in the control group dropped out. At the endpoint (the 6th month), 62 participants in the intervention group and 44 in the control group completed the study (Figure 1).

### 2.2 Clinical assessments

#### 2.2.1 Mini‐international neuropsychiatric interview (MINI) 6.0.0

To be enrolled, participants in both the intervention group and the control group were screened by experienced psychiatrists. The initial clinical diagnoses were subsequently validated by the Mini‐International Neuropsychiatric Interview (MINI) (Kadri et al., 2005; Sheehan et al., 1997). MINI was applied to all our subjects to confirm the clinical diagnosis of major depressive disorder. MINI contained the diagnosis of both current and past mood episodes.

#### 2.2.2 Hamilton Depression Rating Scale (HAMD)‐17

HAMD‐17 was developed in 1960 to assess the severity of depressive symptoms. Most of the HAMD‐17 items were scored on a scale of 0–4 (Park et al., 2014). Cronbach's α score of HAMD‐17 was 0.714, and the κ score was 0.91 (Zhang et al., 2019). We evaluated our patients with HAMD‐17 at baseline, the third month, and the 6th month. Score higher than 7 in HAMD‐17 indicates the presence of depressive symptoms.

#### 2.2.3 Hamilton Anxiety Rating Scale (HAMA)‐14

The HAMA‐14 is one of the most commonly used clinician‐rated measurements of anxiety in studies of depression. HAMA‐14 is rated from 0 to 4 with general guidelines provided for distinguishing the stage‐wise anxiety severity. It is a reliable and valid measure of the severity of anxiety in depressed patients and has become the standard in this field. Score higher than 7 indicates the presence of anxiety symptoms (Wolfgang Maier, Philipp, & Heuser, 1988; Zimmerman et al., 2017).

#### 2.2.4 Revised Social Anhedonia Scale (RSAS)

Social anhedonia is defined as impaired ability to feel pleasure in the interpersonal domain, which is comprised of two factors—social apathy/aversion, and social withdrawal. The Chinese version of the Revised Social Anhedonia Scale is a 40‐binary item self‐rated questionnaire. Higher scores indicate more severe social anhedonia. A series of studies revealed its excellent coefficients exceeding 0.80 (Harvey, Pruessner, Czechowska, & Lepage, 2007; Hu et al., 2018; Rizvi, Pizzagalli, Sproule, & Kennedy, 2016).

#### 2.2.5 Revised Physical Anhedonia scale (RPAS)

Revised Physical Anhedonia scale is developed by the Chapman LJ in 1978 and measures a specific marker of certain endogenous depressions. It is a 61‐binary item self‐rated questionnaire and estimates participants’ inability to experience physical gratification from typically enjoyable stimuli, such as food and situations. This psychometric instrument offers a reliable and valid measure in depressed patients (Kollias et al., 2008). According to many results show that the RPAS had a good positive predictive value for depression and the cutoff of RPAS score was 18 points (Kollias et al., 2008).

### 2.3 Laboratory evaluation

After an overnight fasting period, peripheral venous blood samples (2 ml) were collected from all patients in the morning, samples were sent to the Department of Clinical Laboratory immediately for centrifugation and serum was separated. Serum concentrations of 25‐hydroxyvitamin D (25(OH) D) were measured by radioimmunoassay kits (Immunodiagnostic Systems; Roche).

### 2.4 VD supplementation methods

The subjects with serum 25(OH)D levels ≤75 nmol/L at baseline were randomly divided into an intervention group and control group, patients remained on their current intake (including fish oil) were continued, and the intervention group were given VD 1,600 mg daily supplementation (Qingdao Double Whale Pharmaceutical Co, Ltd.) throughout the 6‐month intervention period (Gowda, Mutowo, Smith, Wluka, & Renzaho, 2015).

### 2.5 Statistical analyses

The *t* tests and chi‐squared test were performed to compare the differences in either continuous or categorical parameters between the two groups. Time differences were explored by repeated‐measure ANOVA and the Tukey post hoc analyses. Then, in order to eliminate the effect of possible confounding variables, linear mixed model analyses and unstructured covariance structure were used to control confounding factors at 6th months. Statistical significance was set when P values < 0.05, two‐sided. All statistical analyses were run on SPSS 22.0 for Windows (IBM Corp).

## 3 RESULTS

### 3.1 Description of sample

The baseline characteristics of participants are shown in Table 1. 158 participants were included in the final analyses. The levels of VD were not different between control (39.1 ± 10.5 nmol/L) and intervention groups (2.1 ± 12.6 nmol/L). No significant differences were observed in age, BMI index, sex and years of education, household income, drinking, smoking, residential city, daytime outdoor time <2 hr, physical activity, sitting time <2 hr, and antipsychotic use between the two groups (all *p* values >.05). Similarly, the HAMD‐17, HAMA‐14, RSAS, and RPAS scores at baseline did not differ between groups (*p* > .05).

**TABLE 1:** Baseline characteristics of the two samples

	Intervention group (*n* = 62)	Control group (*n* = 44)	*F*/χ^2^	*p*
Characteristic variable				
25(OH)D, nmol/L	39.1 ± 10.5	42.1 ± 12.6	1.804	0.182
Age, age	46.3 ± 9.7	43.3 ± 13.7	1.735	0.191
BMI, kg/cm^2^	24.1 ± 4.2	23.6 ± 4.1	0.383	0.537
Blood collection in winter and spring	29(46.8)	22(50.0)	0.107	0.743
Unmarried/divorced	5(8.1)	12(27.3)	7.052	0.27
Male	18(29.0)	10(22.7)	0.526	0.468
City of residence	24(38.7)	20(45.5)	0.487	0.784
Junior high school and below	45(72.6)	27(61.4)	2.017	0.365
Monthly household income <4,000	38(61.3)	28(63.6)	1.818	0.403
Daytime outdoor time: 2 hr < 2 hr	28(53.8)	18(64.3)	0.812	0.368
Drinking	3(5.8)	3(10.7)	0.642	0.423
Smoking	6(11.5)	5(17.9)	0.613	0.434
No physical activity	20(38.5)	8(28.6)	0.783	0.376
Sitting time <2 hr	18(34.6)	8(28.6)	0.303	0.582
No antipsychotic use	5(8.1)	6(13.6)	0.859	0.354
Scale score				
HAMA	18.0 ± 5.7	17.9 ± 7.5	0.001	0.974
HAMD	30.0 ± 7.6	29.2 ± 11.6	0.167	0.684
RPAS	27.2 ± 12.3	27.4 ± 11.7	0.005	0.943
RSAS	16.7 ± 6.0	15.5 ± 6.2	0.692	0.408

Note

Data were displayed with mean ± standard deviation or numbers (%). At baseline, there were no significant differences in general demographic characteristics and scale scores between the intervention group and the control group.

Continuous variables were analyzed by one‐way ANOVA, and categorical variables were analyzed by chi‐square test.

Abbreviations: HAMA‐14, Hamilton Anxiety Rating Scale‐14; HAMD‐17, Hamilton Depression Rating Scale‐17; RPAS, Revised Physical Anhedonia scale; RSAS, Revised Social Anhedonia Scale.

### 3.2 Comparison of psychological scales between the two groups at baseline, 3rd month, and 6th month

**FIGURE 2:**
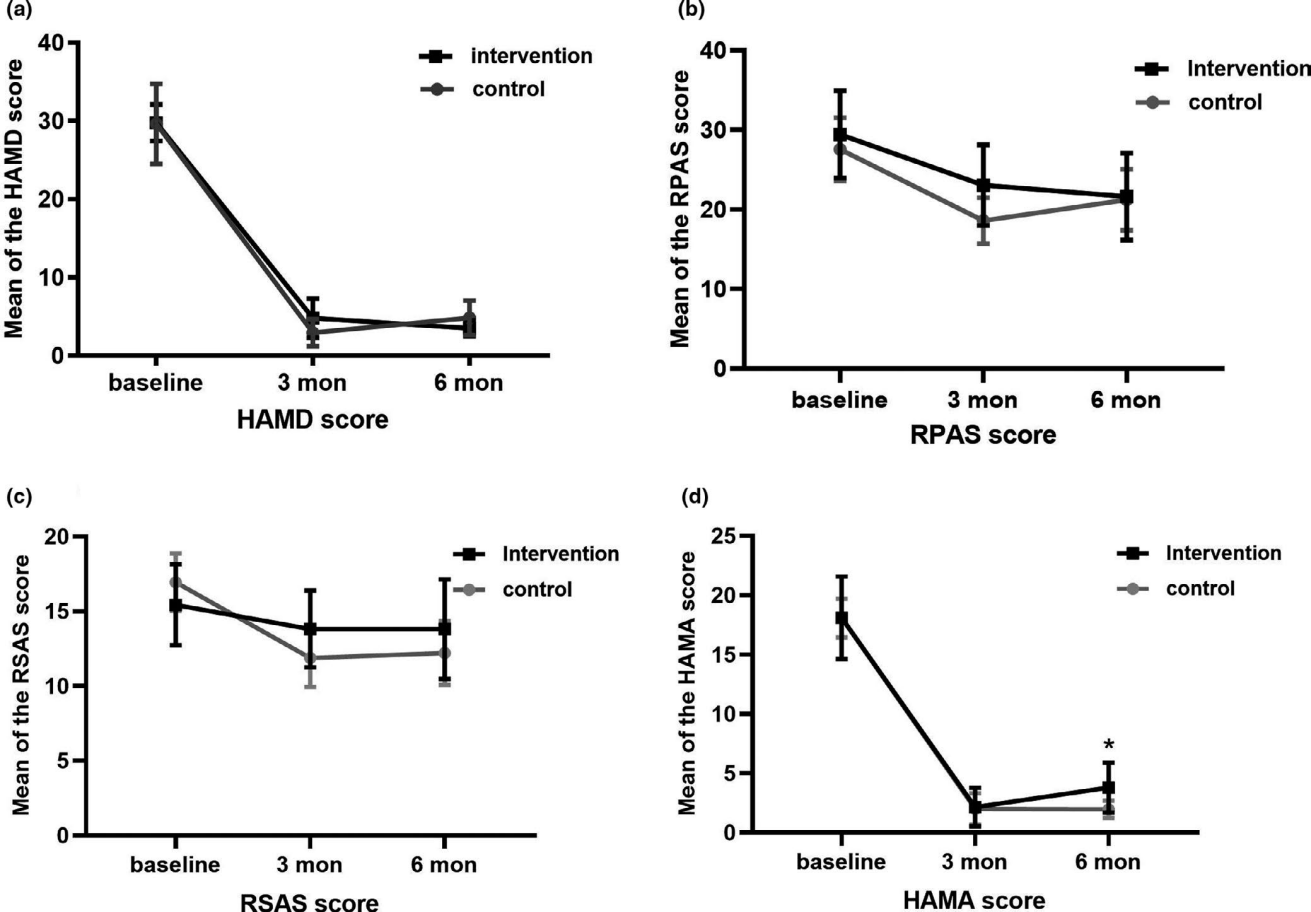
Using the method of one‐way ANOVA to analyze the situation of each evaluation scale after 3rd and 6th monthsof vitamin D intervention. At the 6th month, the difference between HAMA intervention group and control group was statistically significant. HAMA, Hamilton Anxiety Rating Scale, HAMD, Hamilton Depression Rating Scale, RPAS: Revised Physical Scale, RSAS: Revised Social Anhedonia Scale. *p < .05

In the whole cohort, total scores of HAMD‐17 did not differ at baseline, 3rd month, and 6th month in the two groups (Figure 2a, *p* > .05). Additionally, the total scores of RPAS (Figure 2b, *p* > .05) and the total scores of RSAS (Figure 2c, *p* > .05) remained unchanged between the two groups throughout time. There was a significant reduction in the total scores of HAMA‐14 at 6th month between the two groups (intervention group: 8.502 ± 0.483, versus control group: 7.245 ± 0.402, *p* = .041, Figure 2d).

### 3.3 The effect of VD supplementation on the scores of major scales after 6 months

Linear mixed model analyses and unstructured covariance structure were used to control confounding factors after half a year, and all results are presented in Table 2. Linear mixed model analysis results showed the difference in total scores of HAMA‐14 (Model 1: *β* = −2.235, 95% CI = [−3.818, −0.653], *p* = .006) and no difference in total scores of HAMA‐17 [Model 1: *β* = −3.450, 95% CI = (−5.807, −1.093), *p* = .093] in the crude model. Moreover, we found the subitems of HAMA, including BMI [Model 1: *β* = −0.143, 95% CI = (−0.243, −0.042), *p* = .042], day and night changes [Model 1: *β* = −0.065, 95% CI = (−0.281, −0.150), *p* = .018], sense of despair [Model 1: *β* = −0.675, 95% CI = (−4.546, −3.473), *p* = .016], and physical anxiety [Model 1: *β* = −0.843, 95% CI = (−1.627 –0.063), *p* = .045], are significantly different between the two groups after 6 months. In order to avoid the confounding factors, we use unstructured covariance structure to control confounding factors (gender, age, education level, marital status, survey season, smoking, drinking, physical activity time, sitting or lying time, BMI) in the adjusted model. The results shown the differences in total scores of HAMA [Model 2: *β* = −2.414, 95% CI = (−4.455, −0.373), *p* = .021]. The results also demonstrated differences in subitems of HAMA, including BMI [Model 1: *β* = −0.149, 95% CI = (−0.268, −0.030), *p* = .015], sense of despair [Model 1: *β* = −0.546, 95% CI = (−1.051, −0.041), *p* = .034], and physical anxiety [Model 1: *β* = −1.040, 95% CI = (−2.061 –0.019), *p* = .046], are significantly different in the adjusted model.

**TABLE 2:** Comparison between assessment scales after controlling for confounding factors work on the legends

Scale score	Model 1			Model 2		
	*β*	95% CI	*P*	*β*	95% CI	*P*
HAMA	−2.235	−3.818,−0.653	0.006	−2.414	−4.455,−0.373	0.021
Somatization of anxiety	−0.952	−1.694,−0.209	−0.209	−0.701	−1.602,0.200	0.125
Body mass index(BMI)	−0.143	−0.243,−0.042	−0.042	−0.149	−0.268,−0.030	0.015
Cognitive impairment	−0.693	−1.133,−0.252	−0.252	−0.182	−0.509,0.146	0.273
Day and night	−0.065	−0.281,0.15	0.018	0.103	−0.201,0.407	0.499
Slow to respond	−0.801	−1.427,−0.174	−0.174	−0.399	−1.100,0.301	0.259
Sleep disorder	−0.588	−1.061,−0.115	−0.015	−0.355	−0.963,0.227	0.228
Sense of despair	−0.675	−4.546,−3.473	−0.016	−0.546	−1.051,−0.041	0.034
Physical anxiety	−0.843	−1.627,−0.06	−0.045	−1.04	−2.061,−0.019	0.046
Mental anxiety	−0.952	−1.694,−0.209	−0.209	−0.701	−1.602,0.200	0.125
HAMD	−3.450	−5.807,−1.093	−1.093	−1.908	−4.549,0.734	0.154
RPAS	0.892	−3.739,5.524	5.524	0.834	−12.491,4.961	0.789
RSAS	0.250	−2.523,3.023	3.023	−0.414	−4.027,3.198	0.82

Note

Using linear mixed model analyses and unstructured covariance structure to control confounding factors (baseline outcome indicators, gender, age, education level, marital status, survey season, smoking, drinking, physical activity time, sitting or lying time, BMI) at the endpoint of six months of vitamin D intervention.

Abbreviations: HAMA‐14, Hamilton Anxiety Rating Scale‐14; HAMD‐17, Hamilton Depression Rating Scale‐17; RPAS, Revised Physical Anhedonia scale; RSAS, Revised Social Anhedonia Scale.

## 4 DISCUSSION

In this clinical interventional cohort study, we examined the effects of oral 25(OH)D (1,600 IU daily) supplementation for 6 months on depressive patients with VD deficiency (25(OH)D<75 nmol). VD supplementation had no significant impact on depressive symptoms after the 6‐month intervention in the overall cohort. However, there was a significant improvement in anxiety symptoms for patients in the VD supplementation group compared with the control group after adjusting for covariates known to affect VD status. Despite our modest sample size, the findings suggested that high levels of VD may contribute to reduced anxiety symptoms on depressive patients with VD deficiency. Different options for VD deficiency supplementation were available and recommended as augmentation in the treatment of psychiatric disorders.

There is growing evidence showing that VD supplementation may improve the severity of the psychopathology through several potential mechanisms (Gezen‐Ak et al., 2013; Kalueff, Lou, Laaksi, & Tuohimaa, 2004; Yang, Wei, Ju, & Chen, 2019). For example, VD is used to maintain Ca^2+^ homeostasis and this feature is a link between VD deficiency and depression. Another important function of VD interferes with the synthesis of serotonin by the expression of serotonin‐synthesizing gene tryptophan hydroxylase 1 and tryptophan hydroxylase 2, and VD may thus prevent depression by maintaining normal serotonin levels (Berridge, 2017). VD is an important regulator of gene expression in a wide range of cellular functions, and VD receptors were found to mediate several biological pathways including insulin and serotonin, which were associated with depression, anxiety, and other moods (Bertone‐Johnson, 2009; Kelley, Sanders, & Beaton, 2016). These epigenetic alterations that lead to a decline in the expression of key signaling proteins are a feature of many neural diseases including depression. Also, remarkable VD deficiency was shown to be associated with severe more depressive symptoms (Anglin, Samaan, Walter, & McDonald, 2013; Parker & Brotchie, 2011). However, different VD supplementation doses and duration, and levels of serum 25‐hydroxyvitamin (OH)‐D at baseline led to divergent results concerning the effects of psychiatric symptoms. It is worth noting that VD supplementation was more effective in specific populations, such as pregnant women, the elderly, or people living in places with long winters; however, no convincing evidence has yet been found in the causal relationship between VD and depressive symptoms (de Koning et al., 2017; Ju et al., 2013). Moreover, many studies did not adjust or measure for multiple confounders, such as the presence of somatic diseases, physical activity, or psychopharmaceutical state (Jorde & Grimnes, 2019). Furthermore, many studies applied cutoff points on self‐reported psychiatric rating scales as inclusion criterion rather than a clinician‐administered standardized psychiatric interview and therefore endorsed multiple potential biases. Our research found that VD supplementation did not show superiority in the treatment of depression. This result is consistent with the study by Libuda L et al, which is the largest study published so far on VD intervention in depression, and they included 113,769 cases and 208,811 controls and presented a strong but not conclusive argument against a causal relationship between the VD and depression status (Jorde & Kubiak, 2018; Libuda et al., 2019). Studies have also evidenced that there is a minority of depressive patients showing an extreme degree of anhedonia. Anhedonia is a key symptom of MDD and is defined as a reduced capacity to experience pleasurable situations (Treadway & Zald, 2011). RPAS and RSAS were used to evaluate the association between physical anhedonia and social anhedonia with depression in this study, and we did not find any significant difference between hedonic and anhedonic with depressive patients compared to control group in the VD supplementation dose of consumed at 6‐month endpoints, which was in accordance with the previous studies (Kollias et al., 2008).

To the best of our knowledge, many studies focused on the association between serum 25(OH) D levels and depressive symptoms. However, only a few studies examined the relationship between 25(OH) D levels and anxiety symptoms. Animal research showed that the genetic ablation of VD receptors in mice may be associated with altered emotional behaviors and increased anxiety‐like behaviors (Kalueff et al., 2004). In human studies, Armstrong et al demonstrated that fibromyalgia patients accompanied by low levels of VD had higher levels of anxiety and depression symptoms (Armstrong et al., 2007). Similarly, Chao et al reported serum 25(OH) D deficiency showed more symptoms of anxiety and depression on health‐related quality‐of‐life instrument, and Elisa J. de Koning et al found that lower serum 25(OH) D levels were associated with more anxiety symptoms at baseline in older persons. However, after they adjusted it with multiple confounders, this relationship was no longer significant. Moreover, longitudinal studies showed that there was no significant correlation between serum 25(OH) D and anxiety symptoms after three years of follow‐up (de Koning et al., 2017). Notably, our results confirmed that 25(OH) D deficiencies were significantly associated with anxiety symptoms at 6‐month endpoint. The discrepancy among these results gave rise to the need for more research on VD deficiency and anxiety symptoms.

Evidence from experimental and clinical studies had explored the relationship between anxiety symptoms and VD. Dana L. et al found males with anxiety showed a greater trend toward overweight in comparison with controls (Rofey et al., 2009). Dressler et al confirmed that overweight BMI and limited exposure to sun were associated with an increased risk of VD deficiency (Dressler et al., 2016; Pourshahidi, 2014). Our results suggest that BMI is more likely to associate with reduced 25(OH) D concentrations with anxiety symptoms, whatever in the crude model or adjusted model. These findings indicate that BMI is an important factor that should be closely monitored. The feeling of despair may be expressed as hatred and low self‐esteem, and people suffer from a great feeling of despair that can affect their psychological well‐being and lead to outcomes such as depression and anxiety (Thasuk Junprasert, Piasai, & Chaiakkarakan, 2016). In our study also shown was a clear correlation between feelings of despair and VD deficiency, while VD supplementation may help prevent such anxiety symptoms. Given the complexity of depression, it has been shown to be related to multiple neurotransmitter imbalance and declined physical functions and mental capability, and such dysfunctions are the main factors leading to anxiety throughout the course and vice versa (de Koning et al., 2019). Tout results suggest that VD intervention can effectively relieve the physical anxiety of patients, indicating its beneficiary in such patients.

It is worth being noted the several strengths of our study: Most of our patients were in severe depression; the diagnoses were based on semi‐structured interview kit. This paper also examined three dimensions of depressive symptoms—HADM‐17, RSAS, and RPAS, and thus, it provides the comprehensive assessment of the relationship between depression symptoms and VD supplementation conducted to date. On the other hand, there are also several limitations in this study that worth further investigation. First, our study only followed up patients for 6 months, whether the effect of VD could last longer remained unclear. Second, the sample size in this clinical research is relatively smaller; thus, future studies should target on larger‐sized cohorts of depressive patients with VD deficiency. Third, we only implemented one dosage of VD for supplementation, so an ideal or the least effective dosage could not be established through our conclusion. However, indications from our study could provide further optimization of VD supplementation therapy.

In conclusion, we have shown there is no significant correlation between depressive symptoms and VD supplementation, and a clear relationship between low VD levels and high levels of anxiety. Although whether VD deficiency contributes to low mood is still had many confusion, further studies should warrant to address this issue on validation.

## CONFLICT OF INTEREST

None declared.

## AUTHOR CONTRIBUTIONS

Zhu Dao‐min and PengZhu were responsible for study design and manuscript editing. Cuizhen Zhu and Yezhe Lin were responsible for literature searches, statistical analyses, and manuscript writing. Yu Zhang and TingWang were responsible for clinical‐scale assessment data collection. Jiakuai Yu and Qingrong Xia were responsible for clinical blood data collection. All authors have contributed to and have approved the final manuscript.

### Peer Review

The peer review history for this article is available at https://publons.com/publon/10.1002/brb3.1760.

## Data Availability

The data that support the findings of this study are openly available in the Chinese Clinical Trial Registry (http://www.medresman.org.cn/uc/index.aspx, Reference Number: ChiCTR2000030130). We originally included other variables and hypotheses but did not present them in this paper due to space restrictions.
